# Synergistic disruptions in *seuss cyp85A2 *double mutants reveal a role for brassinolide synthesis during gynoecium and ovule development

**DOI:** 10.1186/1471-2229-10-198

**Published:** 2010-09-13

**Authors:** Staci Nole-Wilson, Elizabeth E Rueschhoff, Huda Bhatti, Robert G Franks

**Affiliations:** 1 Department of Genetics, North Carolina State University, Raleigh NC. 27695 USA

## Abstract

**Background:**

The Arabidopsis *SEUSS *(*SEU*) gene encodes a transcriptional adaptor protein that is required for a diverse set of developmental events, including floral organ identity specification, as well as gynoecium, ovule and embryo development. In order to better understand the molecular mechanisms of *SEUSS *action we undertook a genetic modifier screen to identify *seuss-modifier *(*sum*) mutations.

**Results:**

Screening of M2 lines representing approximately 5,000 M1 individuals identified mutations that enhance the *seuss *mutant phenotypic disruptions in ovules and gynoecia; here we describe the phenotype of the *sum63 *mutant and enhanced disruptions of ovule and gynoecial development in the *seu sum63 *double mutant. Mapping and genetic complementation tests indicate that *sum63 *is allelic to *CYP85A2 *(AT3G30180) a cytochrome p450 enzyme that catalyzes the final steps in the synthesis of the phytohormone brassinolide.

**Conclusions:**

Our identification of mutations in *CYP85A2 *as enhancers of the *seuss *mutant phenotype suggests a previously unrecognized role for brassinolide synthesis in gynoecial and ovule outer integument development. The work also suggests that *seuss *mutants may be more sensitive to the loss or reduction of brassinolide synthesis than are wild type plants.

## Background

*SEUSS *(*SEU*) is a member of a family of transcriptional co-regulators that controls a diversity of developmental events in *Arabidopsis thaliana *[1,2]. *SEU *is required for repression of *AGAMOUS *during floral organ identity specification. The SEU protein has been shown to physically interact with members of the MADS domain homeobox transcription factor family as well as other transcriptional co-regulators (LEUNIG (LUG) and LEUNIG_HOMOLOGUE [3-6]). These protein interactions mediate repression of *AG *transcription through the recruitment of a histone deacetylase protein, as well as components of the mediator complex [4,5,7]. These data taken together support a model in which SEU functions as a bridging protein that enables the recruitment of LUG and associated histone deacetylase activities by DNA binding proteins of the MADS domain family. In this model *SEU *is required for repression of *AG *in floral whorls that will give rise to perianth organs where these protein complexes are most active [5].

*SEU *and *LUG *are also required for development of the medial domain of the gynoecium [8,9]. The medial domain of the *Arabidopsis *gynoecium contains the carpel margin meristem, a vital meristem that gives rise to the ovules and other tissues required for female reproductive competence. The effect of *seu *or *lug *single mutants on medial domain development is relatively mild, however both *seu *and *lug *single mutants display a dramatic synergistic interaction with *aintegumenta *(*ant*) mutants. In *seu ant *or *lug ant *double mutants development of the gynoecial medial domain is greatly disrupted resulting in the loss of ovule primordia. These results suggest that *SEU *and *LUG *participate with *ANT *in gene regulation events that are required for the development of the medial gynoecial domain.

*ANT *encodes a DNA binding transcription factor of the *AP2 *gene family that functions during organogenesis [10,11]. *ANT *potentiates organ growth by engendering a competence for cellular divisions during organ development [12,13]. The *ant *single mutants display fewer and smaller lateral organs in both vegetative and reproductive parts of the plant as well as alterations in the development of the ovule integuments [10,11]. The integuments are layers of cells that later form into the seed coat. In the *ant *single mutant both the inner and outer integuments fail to develop properly. *SEU *and *LUG *also play a role in the development of the ovule integuments [1,14]. However, the *seu *and *lug *single mutants display a relatively mild disruption of ovule integument development that is incompletely penetrant.

Brassinosteroid hormones are a class of plant hormones that play a role in a wide variety of developmental processes [15,16]. The two main active brassinosteriod hormones in *Arabidopsis *are castasterone (CS) and brassinolide (BL). The synthesis of these two hormones in *Arabidopsis *requires a cytochrome p450 (cyp450)-type enzyme, *CYP85A2 *(At3G30180) that is rate-limiting for the conversion of 6-deoxyCS to CS and CS to BL [17,18]. However, the phenotype of the *cyp85A2 *mutant is much less severe than that of the brassinosteroid insensitive *bri1 *mutant [19]. This is due in part to the partially redundant activity of a paralogous cytochrome p450 enzyme, *CYP85A1 *(At5G38970) and by the presence of *CYP85A*-independant pathways for the production of CS [17,18].

Here we report synergistic genetic interactions between mutations in the *CYP85A2 *gene and *seu *mutants that affect the development of the gynoecial medial domain and the development of the ovule outer integument. We identified a *cyp85A2 *mutant allele, termed *seuss-modifier 63 *(*sum63*), in a screen for genetic enhancers of the *seu *gynoecial phenotype. Map-based cloning efforts and complementation tests demonstrated that *sum63 *is allelic with existing *cyp85A2 *alleles. The *seu cyp85A2 *double mutants generated in either a Col-0 or L*er *background displayed enhanced disruptions of gynoecial and ovule development. Our results highlight a previously undocumented sensitivity of the *seu *mutants to the reductions in the activity of the brassinosteriod synthesis pathway. This work also points to a role for brassinosteriod hormones in ovule outer integument and gynoecial medial domain development.

## Results and Discussion

**Figure 1:**
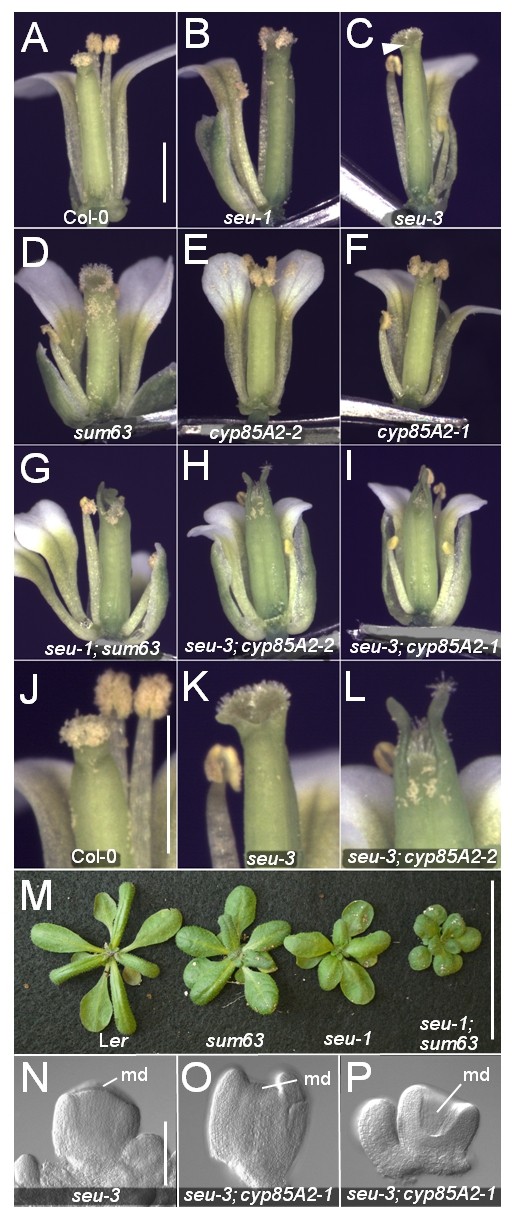
**The *seu cyp85A2 *double mutants condition enhanced gynoecial defects**. Photomicrographs of indicated genotypes: panels A-L, flowers where some sepals and petals have been removed to allow viewing of gynoecia; panel M, rosette morphology. A) Col-0 wild type flower. B) *seu-1 *mutant flower. C) *seu-3 *mutant flower. Note slight split at gynoecial apex (arrowhead). D) *sum63 *single mutant flower. E) *cyp85A2-2 *mutant flower. F) *cyp85A2-1*. Gynoecium splitting is not detected in D-F. G) *seu-1 sum63 *double mutant. H) *seu-3 cyp85A2-2 *double mutant. I) *seu-3 cyp85A2-1 *double mutant. Enhanced splitting at the gynoecial apex is detected in the *seu cyp85A2 *double mutants relative to the respective single mutants. J-L) higher magnification of gynoecial apices shown in A, C and H, respectively. M) Rosette phenotypes. N-P) Nomarski optical images of chloral hydrate cleared stage 7 or early stage 8 gynoecia. N) In *seu-3 *the medial domain (md) extends to apex of gynoecium. At this stage the *seu-3 *mutant gynoecium shown is indistinguishable from wild type (not shown). O) In the *seu-3 cyp85A2-1 *double mutant the extent of the medial domain is reduced. P) Severely effected *seu-3 cyp85A2-1 *gynoecium. Adaxial portions of the medial domain are very reduced resulting in a "hollowed out" gynoecium. Scale bars in A is 1 mm for panels A-I; scale bar in J is 1 mm for images J-L; scale bar in M is 5 cm; scale bar in N is 0.1 mm for N-P.

**Figure 2:**
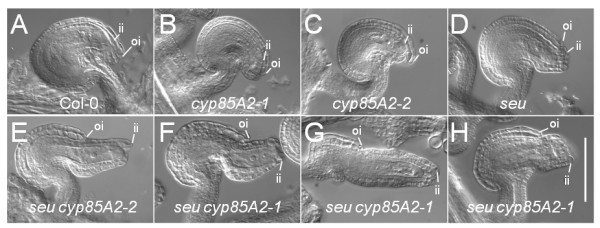
**The *seu cyp85A2 *double mutants condition enhanced outer integument defects**. Photomicrograph (Nomarski optics) of chloral hydrate cleared ovules of the indicated genotypes. Extent of inner (ii) and outer (oi) integument growth is indicated. A) Col-0 wild type. B and C) *cyp85A2-1 *and *cyp85A2-2 *ovules display near wild type morphology. D) *seu-3 *single mutant displays slightly reduced growth of outer integument. E) *seu cyp85A2-2 *ovule. Disruption of outer integument development is greater in the double mutant relative to wild type or either single mutant. F-H) Three examples of *seu cyp85A2-1 *double mutants display varying degrees of outer integument disruption, but are consistently more disrupted than the wild type or single mutants. Scale bar in H is 100 microns for all panels.

The *seu-1 *mutant allele conditions a weak organ identity transformation phenotype that results from the ectopic expression of *AG *[1]. The *seu-1 *mutant also conditions slight splitting of the gynoecial tube and a partially penetrant ovule outer integument defect (Figure 1 and 2). The gynoecial and ovule defects of the *seu-1 *single mutant result in a slight reduction in seed set and gynoecial length (Table 1 and data not shown). We mutagenized *seu-1 *seeds and visually screened the M2 generation for second site mutations that enhanced the sterility and gynoecial splitting of the *seu-1 *mutant (Methods). We uncovered eleven s*euss-modifier *(*sum*) mutations that enhanced the *seu-1 *phenotype. We focused our initial efforts on *sum63 *as it showed a synergistic genetic interaction with *seu *with respect to gynoecial development and female fertility.

**Table 1:** Quantitative phenotypic analysis of *cyp85A2 seu *double mutants

	L*er*	*seu-1*	*sum63*	*seu-1;* *sum63*		Col-0	*seu-3*	*cyp85A2-1*	*cyp85A2-2*	*seu-3;* *cyp85A2-1*	*seu-3;* *cyp85A2-2*
plant height (cm)	12.8 +/- 0.71	8.7^a^ +/- 0.54	9.1^a^ +/- 0.52	4.4^b^ +/- 0.24		14 +/- 0.62	9.4^c^ +/- 0.68	11^c^ +/- 0.57	10^c^ +/- 0.54	6.8^c^ +/- 0.38	N.D.

ovule number per silique	N.D.	N.D	N.D	N.D		53 +/- 2.0	49 +/- 1.4	45^c^ +/- 1.3	45^c^ +/- 1.6	31^d^ +/- 1.1	32^d^ +/- 1.3

^a ^indicates statistically different from L*er *(ANOVA and pair wise Tukey-Kramer HSD; alpha less than 0.05)

^b ^indicates statistically different from L*er*, *seu-1 *and *sum63*

^c ^indicates statistically different from Col-0

^d ^indicates statistically different from Col-0, *seu-3*, *cyp85A2-1 *and *cyp85A2-2*

+/- indicates standard error of the mean

N.D. - not determined

### Molecular identification and phenotypic analysis of *sum63 *single mutants

In order to better characterize the *sum63 *single mutant phenotype in a wild type background, the *seu sum 63 *double mutant identified in the screen was backcrossed to the L*er *parental ecotype three times and *sum63 *single mutant lines were isolated. The *sum63 *single mutants conditioned a moderate reduction in plant height as well as alterations in rosette leaf development (Figure 1M; Table 1). Rosette leaves of the *sum63 *single mutant were darker green and rounder in shape when compared to wild type leaves. The morphology of the *sum63 *flower was similar to wild type except that the stamens were shorter than wild type (Figure 1D). This resulted in a slightly reduced ability of the *sum63 *flower to self fertilize.

We also generated a F2 mapping population by crossing the *seu sum63 *double mutant (L*er *ecotype) to wild type Col-0 plants. The *sum63 *mutation was rough mapped to an interval of chromosome III between ciw11 and T32N15.42 that included the *CYP85A2 *(AT3G30180) gene. Comparison of the *sum63 *single mutant phenotype to the published phenotype for loss-of-function alleles of *CYP85A2 *suggested that *sum63 *might be allelic with *CYP85A2 *[17,18]. We compared the sequence of the *CYP85A2 *genomic DNA from L*er *and *sum63 *individuals in an effort to identify the sequence alteration underlying the *sum63 *allele. We successfully amplified and sequenced the first 1274 nucleotides of the *CYP85A2 *gene sequence (transcriptional start site as +1) as well as a 3' portion of the *CYP85A2 *gene from position +2427 into the 3'untranslated region (See Table 2 for primer sequences). We found no changes in the sequence of these regions in the *sum63 *mutant relative to the sequences we derived from L*er *DNA. However, we were unable to amplify the intervening portions of the *CYP85A2 *gene from the *sum63 *mutants, while these same regions were successfully amplified from L*er *individuals. Furthermore, oligonucleotide primers that spanned the intermediate region (i.e. between +1274 and +2427) also failed to generate amplicons from the *sum63 *genomic DNA. These results suggested the presence of a genomic rearrangement in *sum63 *individuals that disrupts the *CYP85A2 *gene sequence. Complementation tests between the *sum63 *allele and the previously characterized *cyp85A2-1 *and *cyp85A2-2 *alleles [18,20] revealed that *sum63 *was allelic with these *cyp85A2 *alleles (data not shown). Thus we have renamed the *sum63 *allele *cyp85A2-4 *(Table 3). Characterization of the T-DNA insertion sites in *cyp85A2-1 *and *cyp85A2-2 *individuals confirmed disruption of this gene in these lines (Methods). The *cyp85A2-1 *allele has been previously reported as a null allele based on a failure to detect transcript in RT PCR assays [17]. The phenotypes of the *cyp85A2-1, cyp85A2-2 *and *cyp85A2-4 *alleles are similar suggesting that they are all strong loss-of-function alleles (Figure 1).

**Table 2:** Sequences of primers used for amplification and sequencing of the *CYP85A2 *gene

primer name	sequence
AT3G30180-F1	TAAACAACGCCACACACACC

AT3G30180-613R	CAACGAGCCTCTCATTAGCC

AT3G30180-445F	TGGTTGCCCAACAATAGTCTC

AT3G30180-1274R	TCCCACAACAAGCTTGAAAA

AT3G30180-2427F	TTTGGTGCTCTTGTGTTTTG

AT3G30180-3UTRR	CATTGCAAGTAGGCCCAAAT

AT3G30180-2938R	TTCCATTTTCTTCTTCTCTCTTTCTC

**Table 3:** *CYP85A2 *mutant alleles described in this study

Allele	Other Designation	Allele Disruption	Reference
*cyp85A2-1*	*salk_056270*	T-DNA insertion in CDS at amino acid 45	[17,18]

*cyp85A2-2*§	*salk_129352*	T-DNA insertion in CDS at amino acid 302	[17,18]

*cyp85A2-4*	*sum63*	uncharacterized rearrangement	This study

§ this allele (*salk_129352) *has been designated *cyp85A2-2 *by Nomura et al., 2005. The allele designated *cyp85A2-2 *in Kim et al., 2005 is a different allele (*salk_068754*) that was designated *cyp85A2-3 *by Nomura et al.

### The *seu cyp85A2 *double mutant conditions enhanced disruptions of gynoecial and ovule development

To further characterize the *seu cyp85A2 *double mutant phenotype we created and analyzed the following double mutants: *seu-3 cyp85A2-1*, *seu-3 cyp85A2-2 *(both in the Col-0 background) and *seu-1 cyp85A2-4 *(L*er *background). We did not detect an enhancement of the homeotic transformations previously reported for the *seu-1 *allele [1] in the *seu cyp85A2 *double mutants. However, all three *seu cyp85A2 *double mutant combinations displayed enhanced defects in ovule and gynoecial development. Double mutants conditioned enhanced splitting of the apex of the gynoecium relative to the single mutants (Figure 1). The *cyp85A2 *single mutants did not display splitting of the gynoecial apex and splitting in the *seu *single mutant was mild and rarely observed in the early arising flowers. In contrast, the gynoecial apex in the *seu cyp85A2 *double mutants was nearly always split and extended horn like protrusions of the valves were observed. The splitting of the apex and the horn-like protrusions may be the result of a reduction in the growth of the medial domain of the gynoecium. Analysis of earlier stage gynoecia indicate that even as early as floral stage 7 or 8 [21] the medial domain of the gynoecium appears retarded in its growth relative to the lateral or valve domains (Figure 1N-P). The *seu cyp85A2 *double mutants also display a significant reduction in the number of ovule primordia initiated relative to wild type and either single mutant (Table 1). Kim et al. have previously reported that over-expression of *CYP85A2 *conditions an increased number of seeds per silique, further suggesting a role for *CYP85A2 *in the development of ovules from the gynoecial medial domain [17].

**Figure 3:**
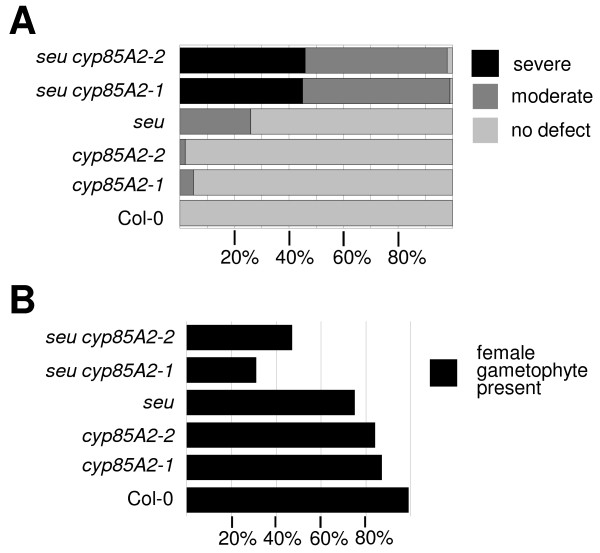
**Analysis of outer integument and female gametophyte phenotypes in *seu cyp85A2 *double mutants**. A) Bar graph depicting the percentage of ovules displaying each phenotypic class for the indicated genotypes. Outer integument defects were classified into three phenotypic classes: "severe defect", less than 50% of ovule covered by outer integument; "moderate defect", between 50 and 90 percent of ovule covered by outer integument; "no defect", greater than 90% of ovule covered by outer integument. B) Bar graph depicting the percentage of ovules displaying a morphologically wild type female gametophyte for indicated genotypes.

The *seu cyp85A2 *double mutants also conditioned an extreme loss of fertility as these double mutants did not generate viable seeds upon self-fertilization. Our analysis of ovule defects indicated the ovule developmental defects of the double mutant were enhanced relative to either single mutant. The double mutant ovules displayed a reduced growth of the outer integument relative to either single mutant (Figs. 2 and 3). In severe cases the outer integument failed to develop, resulting in a somewhat orthotropic ovule morphology (Figure 2G). Although brassinolide has not previously been reported to play a role in outer integument development, the double mutant phenotype suggests a requirement for the *cyp85A2 *gene for outer integument development in the *seu *mutant background. It is likely that the loss of *SEU *sensitizes the ovule to disruptions in the levels of brassinolide.

We also noted defects in the development of the female gametophytes within the *seu cyp85A2 *ovules (Figure 3). Often the female gametophyte was missing or failed to reach a mature morphology with a recognizable set of gametophyte cells. As we did not observe segregation distortion with these alleles, it is most likely that the defects in female gametophyte development are an indirect result of the development of the sporophytic tissue of the ovule and not due to a requirement for *SEU *and *CYP85A2 *activity in the female gametophyte directly.

### Levels of *CYP85A2 *transcript are reduced in seuss mutant inflorescence samples

We examined the steady state levels of transcript accumulation for the *CYP85A2 *and *SEU *transcripts in wt (Col-0), *seu-3 *and *cyp85A2-1 *mutant inflorescences. In the *seu-3 *mutant tissue the level of the *CYP85A2 *transcript was significantly less (41% of wild type) than that detected in wild type inflorescence samples (Table 4). These data suggest that *SEU *activity may be required for wild type levels of *CYP85A2 *transcript accumulation in the inflorescence. No statistically significant difference in the expression of *SEU *transcript was detected between the wild type and the *cyp85A2 *inflorescences tested. Thus *CYP85A2 *does not appear to be required for expression of the *SEU *transcript. The *cyp85A2-1 *allele has been previously reported as a null allele, yet we detected a low level of expression of the *CYP85A2 *transcript in the *cyp85A2-1 *inflorescences. However, the *cyp85A2-1 *allele is still likely a null or near null allele based on the site of the T-DNA insertion that is expected to truncate the *CYP85A2 *protein product after just 45 amino acids. Thus it seems unlikely that the synergistic enhancement of the *seu cyp85A2 *double mutants is entirely conditioned by a *seu*-mutant dependant reduction of *CYP85A2 *transcript.

**Table 4:** qRT-PCR quantification of mean expression levels^§ ^of *CYP85A2 *and *SEU *transcripts in wt, *seu *and *cyp85A2 *mutants.

	wt (Col-0)	*seu-3*	*cyp85A2-1*
*CYP85A2*	0.29 +/- 0.03	0.12^a^ +/- 0.01	0.12^a^ +/- 0.02

*SEU*	0.43 +/- 0.02	0.12^a^ +/- 0.01	0.39 +/- 0.05

^a ^indicates statistically different from Col-0

(ANOVA and pair wise Tukey-Kramer HSD; alpha less than 0.05)

+/- indicates standard error of the mean

^§ ^Normalized to *ADENOSINE PHOSPHORIBOSYL TRANSFERASE *(AT1G27450)

We propose that additional genes are misregulated in the *seu *mutant background and that these disrupted gene regulation events contribute to the phenotypic enhancement. We speculate that these genes might lie in the brassinosteroid synthesis pathway or might lie in parallel pathways that support common cellular responses (e.g. cell division or cell expansion) in the ovule or the gynoecial medial domain. *SEU *is required for proper response to the phytohormone auxin and altered auxin signaling may in part condition the disruption of gynoecium medial domain development seen in *seu ant *double mutants [2,22]. Given the extensive overlap of brassinosteroid-responsive and auxin-responsive genes and the documented requirement of brassinosteroids for transcriptional responses to auxin [23-28], it is possible that the enhanced *seu cyp85A2 *double mutant phenotypes result from the combined weakening of brassinosteroid- and auxin-dependant signaling pathways.

## Conclusions

Our screen for second site genetic modifiers of the *seu *mutant gynoecial and ovule phenotypes has identified *cyp85A2 *as a genetic enhancer of the *seu *mutant. These results suggest that brassinolide hormones play a previously unappreciated role in the development of the outer integument of the ovule and the gynoecial medial domain. They also suggest that loss of *SEU *activity may sensitize ovule and gynoecial development to the loss of brassinosteroid hormones. The *seu *mutant background thus may represent a sensitized genetic background to identify additional regulators of gynoecial and ovule function.

## Methods

### Microscopic and morphometric analysis

For chloral hydrate clearing inflorescences were fixed in ethanol:acetic acid (9:1) for two hours at room temperature, washed in 90% ethanol (two times) and then cleared overnight at room temperature in either chloral hydrate (Sigma) (2.5 g dissolved in 1 ml of 30% glycerol) or Hoyer's solution (70% chloral hydrate, 4% glycerol, 5% gum arabic (Sigma)) [29]. Gynoecia were then dissected and mounted on slides in Hoyer's solution, and examined on Axioscop2 microscope (Zeiss) with Nomarski optics. Ovule counts were made in chloral hydrate cleared stage 9-12 gynoecia. Estimations of outer integument defects and female gametophyte development were made from mature ovules or young seeds observed in stage 12 -14 flowers. Images were captured with a micropublisher 5.0 RTV digital camera and Q capture software (Q Imaging, Surrey, BC, Canada). Cropping and contrast adjustment of images was done in Adobe Photoshop CS2 (Adobe Software). Plant heights were measured manually when plants were at an equivalent developmental age as determined by the number of post-abscission siliques observed on the primary shoot. Statistical analysis was carried out in JMP7 (SAS Institute Incorporated) using ANOVA followed by pair wise comparisons with a Tukey-Kramer HSD test and an alpha value cutoff of 0.05.

### Genotyping and T-DNA insertion site mapping

The T-DNA insertion site was mapped for salk_056270 (*cyp85A2-1*) in At3g30180 within the end of exon 1 in the codon encoding glycine 45. The asterisk (*) indicates the insertion site within the genomic DNA sequence: 5'GCCAATATTTGGTGA*AACGACTGAGTTTCT3'). The T-DNA insertion site was mapped for salk_129352 (*cyp85A2-2*) within the end of exon 4 in the codon encoding glutamic acid 302. The asterisk (*) indicates the insertion site within the genomic DNA sequence: 5'AGCTCTTGAAGAACT*CAGAGTATGTACTT3'.

The oligonucleotide pairs: SALK_056270 LP (5'GAATTTCGTGCTGAAAATTGC3'), SALK_056270 RP (5'ACCCGAGATTCAGATTCAATG3'); and SALK_129352 LP (5'CGTAAATTCTCCAACCTTTTGG3'), SALK_129352 RP (5'TTGTTGTGGGAACTCTATCGG3') were used with oligo LBb1 (5'GCGTGGACCGCTTGCTGCAACT3') for genotyping and insertion site mapping.

### qRTPCR analysis

For analysis of transcript abundance, inflorescences (inflorescence meristem through floral stage 12) were collected, frozen in liquid nitrogen and ground in microcentrifuge tubes. RNA extraction, cDNA synthesis, and qRT-PCR were performed as previously described [8]. A single qRTPCR run contained four biological replicates, and each biological replicate was assayed in triplicate. Results shown in Table 4 are the mean expression of the indicated gene normalized to *ADENOSINE PHOSPHORIBOSYL TRANSFERASE *(At1g27450). Results are averages and standard error of the mean for four biological replicates. Statistical analysis of differences of means was carried out in JMP7 (SAS Institute Incorporated) using a Tukey-Kramer HSD test and a p value cutoff of 0.05. Sequences of the oligonucleotides used for qRT-PCR analysis: At3G30180-1440/1441F (5'GGAGGTGGAGTTAGGCTTTGCCC3') and At3G30180-1440/1441R - (5'TCTTCTCCATTCTCTTCCCATCTAT3')

### Mutagenesis

Approximately 5,000 homozygous *seu-1 *seeds were imbibed overnight in 4 mM ethyl methane sulfonate in a 50 ml conical tube. Seeds were then rinsed several times and planted to soil. Pools of M2 seeds were collected from approximately fifteen M1 plants. M2 pools were screened for enhanced gynoecial defects or female sterility.

### Mapping of sum63 mutation and sequencing of *CYP85A2 *genomic DNA from the sum63 mutant

Pollen from *sum63 seu-1 *double mutants (in the L*er *background) was used to pollinate Col-0 plants. Resulting F2 plants were visually screened and genomic DNA was prepared from leaves of 53 plants that displayed the *sum63 *phenotype. The *sum63 *mutation was rough mapped between ciw11 and T32N15.42 on the third chromosome. We estimated the percentage recombination between the ciw11 marker and the *sum63 *mutation was 9.8% and between T32N15.42 and *sum63 *was 10.2%. The oligonucleotide pairs AT3G30180-F1 and AT3G30180-613R as well as AT3G30180-445F and AT3G30180-1274R were used to amplify and sequence the first 1274 nucleotides (relative to transcriptional start site as position +1) of the *CYP85A2 *genomic DNA sequence from L*er *and *sum63 *samples (See Table 2 for primer sequences). The oligonucleotide pair AT3G30180-2427F and AT3G30180-3UTR was successfully used to amplify and sequence the 3'-most portions. Several primer pairs expected to hybridize to the intervening portions of the *CYP85A2 *gene, as well as primer pairs that were expected to span the intervening region (i.e. AT3G30180-445F and AT3G30180-2938R) did not generate amplicons from the *sum63 *DNA while they did yield amplicons from the L*er *DNA samples.

## List of abbreviations used

*SEUSS *(*SEU*): *seuss-modifier *(*sum*); (CYP): cytochrome p450; (CS): castasterone; (BL): brassinolide; (L*er*): Landsberg *erecta*; (Col): Columbia; (*LUG*): *LEUNIG*; (*ANT*): *AINTEGUMENTA*;

## Authors' contributions

SNW, EER, HB, and RGF all contributed to data collection and analysis. RGF devised the screen and wrote the paper. All authors have read and approved the final manuscript.
